# lncRNA MEG3 Promotes PDK4/GSK-3*β*/*β*-Catenin Axis in MEFs by Targeting miR-532-5p

**DOI:** 10.1155/2023/3563663

**Published:** 2023-02-01

**Authors:** Yuan-Yuan Yang, Yi-Xuan Deng, Xin-Tong Yao, Hong-Hong Luo, Wen-Ge He, Xuan-Ling Cao, Rong-Chun Chen, Bai-Cheng He, Hai-Tao Jiang, Jing Wang

**Affiliations:** ^1^Department of Blood Transfusion, The First Affiliated Hospital of Chongqing Medical University, Chongqing 400016, China; ^2^Chongqing Key Laboratory of Biochemistry and Molecular Pharmacology, Chongqing Medical University, Chongqing 400016, China; ^3^Department of Pharmacology, School of Pharmacy, Chongqing Medical University, Chongqing 400016, China; ^4^Department of Orthopedics, The Second Affiliated Hospital of Chongqing Medical University, Chongqing 400010, China

## Abstract

Studies reported the positive and negative osteogenic effects of MEG3 in mesenchymal stem cells (MSCs). This study aims at clarifying the osteogenic potential of MEG3 and the underlying mechanism. Bone morphogenetic protein 9- (BMP9-) transfected MSCs were recruited as an osteogenic model *in vitro*, and ectopic bone formation were used *in vivo* to explore the effect of MEG3 on osteogenesis. We found that overexpression of MEG3 facilitated BMP9-induced osteogenic markers, ALP activities, and matrix mineralization. However, knockdown of MEG3 attenuated BMP9-induced osteogenic markers. MEG3 increased the phosphorylation of GSK-3*β* and the protein level of *β*-catenin. Pyruvate dehydrogenase kinase 4 (PDK4) can also combine with GSK-3*β* and increase the latter phosphorylation. Moreover, MEG3 increased the mRNA level of PDK4. The ceRNA analysis showed that MEG3 may regulate the expression of PDK4 via microRNA 532-5p (miR-532-5p). The MEG3-enhanced GSK-3*β*/*β*-catenin axis can be attenuated by miR-532-5p, and miR-532-5p inhibitor partly rescued endogenous PDK4 and MEG3-mediated expression of PDK4. MEG3 may potentiate PDK4 and GSK-3*β*/*β*-catenin by inhibiting miR-532-5p.

## 1. Introduction

Long noncoding RNAs (lncRNAs) are emerging as important regulators in growth, development, and tumorigenesis. In the nucleus, they regulate gene expression networks by controlling nuclear architecture and transcription; in the cytoplasm, they also modulate mRNA stability, translation, and posttranslational modifications [1–3]. Maternally expressed gene 3 (MEG3) is a kind of lncRNA located on chromosome 14q32.3 [4]. Regarded as a tumor suppressor, previous studies mainly focus on the antitumor effect of MEG3. It has been reported that MEG3 is downregulated in tumoral tissues compared with nontumoral tissues. In addition, overexpression of MEG3 attenuates the proliferation and induces apoptosis in different tumor cell lines, such as squamous cell carcinoma and colorectal cancer [5, 6]. In recent years, an increasing number of studies reveal that MEG3 is involved in osteogenic differentiation of mesenchymal stem cells (MSCs). It has been reported that upregulation of MEG3 activates BMP4-mediated osteogenesis [7], while inhibition of MEG3 shows reduced BMP4 level and the phosphorylation of Smad1/5/8 [8]. However, the effect of MEG3 on other osteogenesis-related pathways such as Wnt/*β*-catenin and Notch remains unknown.

MicroRNAs (miRNAs) are short, noncoding RNAs that function by destabilizing and/or suppressing the translation of their target RNAs [9]. Accumulating evidence has shown that lncRNAs play an important role in osteogenic differentiation by acting as a competing endogenous RNA (ceRNA) for miRNAs sponge [10–12]. Studies have shown that miRNA 532-5p (miR-532-5p) correlate significantly with lumbar spine bone mineral density, but the exact effect and possible mechanism awaits further exploration [13].

The mitochondrial enzyme pyruvate dehydrogenase complex (PDC) irreversibly decarboxylates pyruvate to acetyl coenzyme A (acetyl CoA) and controls the following tricarboxylic acid (TCA) cycle [14]. PDC is regulated by a phosphorylation-dephosphorylation cycle, in which the pyruvate dehydrogenase kinase (PDK) phosphorylates and inactivates the complex [15]. As one of the four PDK isozymes (PDK1, 2, 3, and 4), previous studies of PDK4 mainly focus on the regulation of energy metabolism and miRNA-mediated cancer research [16–18]. However, an increasing number of reports reveal that PDK4 play a pivotal role in vascular calcification and osteogenic differentiation [19]. During osteogenic induction in vascular smooth muscle cells, the expression of PDK4 is increased [20]. Upregulation of PDK4 promotes vascular calcification by increasing the phosphorylation of Smad1/5/8 and other osteogenic markers [21]. However, the relationship with miRNAs and the osteogenic effect of PDK4 remain to be further investigated.

Glycogen synthase kinase-3*β* (GSK-3*β*) is a kind of serine-threonine kinases. As a member of canonical Wnt/*β*-catenin pathway, the function of GSK-3*β* is to regulate the translocation of *β*-catenin into nucleus. When the Wnt receptor Frizzled/LRP is not engaged by ligands, GSK-3*β* continuously phosphorylate Axin-bound *β*-catenin at Thr41, Ser37, and Ser33 [22], leading to the degradation of *β*-catenin by E3 ubiquitin ligase *β*-TrCP [23]. When the Frizzled/LRP receptor is engaged by Wnt ligands, the phosphorylated LRP receptor may act to inhibit GSK-3*β* directly and facilitate the phosphorylation and degradation of GSK-3*β* [24]. At the same time, the stabilization of *β*-catenin is promoted, and free *β*-catenin level rapidly increases. Finally, the accumulated *β*-catenin can translocate into nucleus and activate target genes [25, 26], such as LEF/TCF.

In the present study, we confirmed the positive osteogenic effect of MEG3, and elucidated the possible mechanism underlying this effect, which was closely related to the inhibition of miR-532-5p and subsequent activation of PDK4/GSK-3*β*/*β*-catenin axis.

## 2. Materials and Methods

### 2.1. Cell Culture and Chemicals

C3H10T1/2, C2C12, and HEK-293 cell lines were purchased from ATCC (Manassas, VA, USA). Cells were cultured in dishes, flasks, or plates with Dulbecco's modified Eagle's medium which contained 10% fetal bovine serum (FBS), streptomycin (100 *μ*g/mL), and penicillin (100 U/mL). These dishes, flasks, and plates were cultured in 37°C incubators with 5% CO_2_. Primary antibodies against Runx2 (sc-390715), OPN (sc-21742), *β*-catenin (sc-7963), GSK-3*β* (sc-81462), p-GSK-3*β* (sc-81496), and PDK4 (sc-518103) were bought from Santa Cruz Biotechnology (CA, USA); GAPDH (10494-1-AP) was purchased from Proteintech (Wuhan, China); COXIV was ordered from Cell Signaling Technology (Boston, USA). Dylight 594 conjugated secondary antibody (A23410) was bought from Abbkine (Chinese branch). Alizarin Red S (ST1078) and Alkaline Phosphatase Assay Kit (C3206) were obtained from Beyotime (Shanghai, China).

### 2.2. Construction of Recombinant Adenoviruses

The recombinant adenoviruses used for knockdown or overexpression in this study were constructed based on Ad-Easy system [27, 28]. Briefly, the coding regions of green fluorescent protein (GFP), red fluorescent protein (RFP), BMP9, MEG3, and PDK4 were cloned into the shuttle vectors, so were the MEG3 and PDK4 siRNA oligo fragments. Then, these above plasmids were linearized by restriction endonuclease. Subsequently, they were transformed into competent AdEasier cells which contain the plasmid backbone of adenovirus. Finally, the recombinant plasmids were cut by PacI and transfected into HEK-293 cells, followed by culturing for 14 days. The recombinant adenoviruses were designated as Ad-GFP, Ad-RFP, Ad-MEG3, etc. Among them, Ad-GFP and Ad-RFP were used as vector control to track the virus without target gene expression.

### 2.3. Isolation of Mouse Embryonic Fibroblasts (MEFs)

MEFs were isolated from postcoitus day 12.5 mice embryos as previously described [29]. Briefly, the embryo was dissected, sheared by an 18-gauge syringe in 1 mL 0.25% trypsin. Then, the tissues were incubated at 37°C for 15 min with gentle shaking. Subsequently, 10 mL DMEM containing 10% FBS was added to deactivate the trypsin. Finally, the dissected cells were plated in dishes and incubated at 37°C, 5% CO_2_ for 24 h. All MEFs used in this study were within five passages.

### 2.4. RNA Sequencing

MEFs and osteogenesis-resistant (OR) MEFs were seeded in dishes, respectively. After adherence, the MEFs and OR MEFs were washed twice with PBS and harvested on ice with TRIZOL agent (Invitrogen). The samples were sent to Novogene Biotech Co. Ltd. (Beijing, China) for RNA-seq analysis [30]. Briefly, the sequencing libraries were generated by NEBNext UltraTM RNA Library Prep Kit for Illumina (NEB) following the manufacturer's instructions. The quality of library was evaluated by the Agilent Bioanalyzer 2100 system. Then, the profiles of gene expression of the groups were processed using the Illumina Hiseq platform, followed by the data analysis of genes that differentially expressed. Another RNA sequencing was performed using MEFs as previously described; the groups were Ad-GFP, Ad-BMP9, Ad-siMEG3, Ad-BMP9 + Ad-siMEG3.

### 2.5. ceRNA Network Construction

The ceRNA network was constructed based on the target gene of miRNA and the expression correlation. The TargetScan v7.2, miRanda v3.3a, and RNAhybrid V2.1.2 were used to predict the target genes of miRNA. Given the same miRNA target gene share network, the expression Pearson correlation cutoff (≥ 0.9) for each pair will select ceRNA network edges. The lncRNA-miRNA pairs and miRNA-mRNA pairs were screened to construct the lncRNA-miRNA-mRNA network and visualized by Cytoscape V3.8.2 software.

### 2.6. miRNA Mimic and Inhibitor

The miR-532-5p mimic, miR-532-5p inhibitor, and their corresponding control were bought from GeneBio (Shanghai, China). They were transfected with a final concentration of 50 nM into MEFs using Lipofectamine 2000 (Life Technology, USA) according to the manufacturer's instructions. The sequence for miR-532-5p mimic, CAUGCCUUGAGUGUAGGACCGU, ACGGUCCUACACUCAAGGCAUG; miR-532-5p mimic NC, UCACAACCUCCUAGAAAGAGUAGA, UCUACUCUUUCUAGGAGGUUGUGA; miR-532-5p inhibitor, ACGGUCCUACACUCAAGGCAUG; miR-532-5p inhibitor NC, UCUACUCUUUCUAGGAGGUUGUGA.

### 2.7. Dual Luciferase Reporter

The sequences of MEG3-WT, MEG3-MUT, miR-532-5p NC, and miR-532-5p were previously inserted into the vector psiCHECK2, respectively. Then, the recombinant vectors were transfected into HEK-293 T cells with Lipofectamine 2000 according to the group division (MEG3-WT + miR-532-5p NC, MEG3-WT + miR-532-5p, MEG3-MUT + miR-532-5p NC, and MEG3-MUT + miR-532-5p). The cells from each group were collected after 48 h and subjected to Dual-Luciferase® Reporter Assay System (E1910, Promega, Chinese branch) following the manufacturer's instructions.

### 2.8. Coimmunoprecipitation

At the designed time point, cells were washed twice with PBS and lysed on ice using RIPA lysis buffer (Solarbio, China), phosphatase, and protease inhibitors. The cell lysates were then centrifuged (12000 rpm for 10 min), and the supernatants were collected. The magnetic beads of protein G (NEB, Chinese branch) were washed with 30 *μ*L lysis buffer and supernatants for preclearance. The supernatants were incubated with primary anti-PDK4 antibody or IgG overnight (4 degrees Celsius). Next day, the washed beads were added to complexes separately, followed by shaking for 3 h (4 degrees Celsius). Then, these complexes containing beads were collected with the magnetic stand, followed by RIPA buffer wash for 5 times. Finally, the obtained proteins were boiled and subjected to western blot analysis. Primary anti-p-GSK-3*β* antibody was used for western blot detection.

### 2.9. Osteogenesis-Resistant (OR) MEFs Preparation

OR MEFs were constructed by small RNA library screening and ectopic bone formation. Briefly, MEFs were transfected with retrovirus containing the small RNA library, followed by BSD screening for the positively transfected MEFs. Then, the positively transfected MEFs were transfected with Ad-BMP9 for several rounds, and the living cells were screened out for ectopic bone formation. Subsequently, the ectopic bones were retrieved, and the uncalcified cells were separated for the second round of BMP9 transfection and ectopic bone formation. After the third round of screening, the uncalcified cells were retrieved and regarded as OR MEFs.

### 2.10. Osteogenic Inductive Medium (OIM) Preparation

OIM contains dexamethasone, sodium *β*-glycerophosphate, and L-ascorbic acid. Sodium *β*-glycerophosphate and L-ascorbic acid were dissolved in DMEM and filtered using a 0.2-*μ*m filter. Dexamethasone was dissolved in DMSO. The obtained solutions were added to DMEM containing 10% FBS. The final concentration of dexamethasone was 10 nM; sodium *β* glycerophosphate was 10 mM, and L-ascorbic acid was 50 *μ*g/mL [30]. For osteogenic differentiation, the cells were seeded in 6-well or 24-well plates with DMEM and transfected with different adenoviruses after adhesion. After 36 h, the medium was replaced by OIM, which was renewed twice a week until the final ALP or Alizarin Red S staining.

### 2.11. RNA Extraction and Real-Time Quantitative Polymerase Chain Reaction

At the preset timepoints, total RNA was extracted using TRIZOL reagent (Invitrogen, USA), purified, and used for reverse transcription (RT) and cDNA generating. The resultant cDNA was diluted 5-10 folds for qPCR assay. The expression of the housekeeping gene GAPDH was used to normalize the PCR results. MEG3, Runx2, DLX-5, etc. were regarded as the target genes. The primer sequences used in this study were presented in Table 1.

### 2.12. Protein Extraction and Western Blot

At the designed timepoint, the cells in 6-well plates were washed twice with phosphate buffered saline (PBS) and lysed with 300 *μ*L lysis buffer on ice. Subsequently, the lysate was transferred into 1.5 mL Eppendorf tubes, followed by the addition of 15 *μ*L *β*-mercaptoethanol and 75 *μ*L loading buffer in each tube. Then, the tubes were bathed in boiling water for 15 min and stored in -20°C or -80°C. The samples were separated by sodium dodecyl sulfate polyacrylamide gel electrophoresis (SDS-PAGE), and the gels containing target protein were transferred to polyvinylidene difluoride (PVDF) membranes. The membranes were bathed in 5% bovine serum albumin (BSA) for 1 h at room temperature and then incubated in the primary antibody solutions at room temperature for 1.5 h or at 4°C overnight. The membranes were washed five times with Tris-buffered saline Tween20 (TBST) and incubated in corresponding secondary antibodies (Beyotime, Shanghai, China) for 0.5 h at room temperature. Finally, the membranes were washed five times with TBST, and the images of the target bands were taken using SuperSignal West Pico Chemiluminescent Substrate (SWPCS) (Thermo Scientific, IL, USA). The primary antibodies were diluted in 5% BSA with a ratio of 1 : 1000; the secondary antibodies were diluted 1 : 3000 in TBST.

### 2.13. Alkaline Phosphatase Staining

At the designed timepoint, the cells in 12-well or 24-well plates were washed twice with PBS and stained with the working solution (200 *μ*L/well) from Beyotime Alkaline Phosphatase Assay Kit for 15 min in the dark. Briefly, the working solution contained BCIP, NTB, and ALP buffer, at a ratio of 33 *μ*L : 66 *μ*L : 10 mL. These plates were dried and scanned for data analysis. For microscopic images, the wells were photographed using a fluorescence microscope (Nikon, Japan). These results were repeated in at least three independent experiments.

### 2.14. Alizarin Red S Staining and Quantification

At the corresponding timepoint, the cells in 6-well or 24-well plates were washed twice with PBS and fixed with 4% paraformaldehyde for 10 min. Then, the wells were washed with pH 4.2 PBS gently and stained with 0.4% Alizarin Red S staining solution. Subsequently, the plates were dried and scanned. For microscopic images, each well was photographed using a fluorescence microscope (Nikon, Japan). Finally, each well was bathed in 10% acetic acid, and the absorbance at 405 nm was detected using a microplate reader as previously described [31, 32]. These data were standardized to corresponding control. These results were repeated in at least three independent experiments.

### 2.15. Immunofluorescence Staining and Confocal Laser Scanning

At the designed timepoint, the cells in 48-well plates were washed with PBS and fixed with 4% paraformaldehyde. Subsequently, the cells were bathed in 0.3% TritonX-100, followed by PBS wash for three times. Then, the cells were incubated with goat serum (Beyotime, Shanghai, China) at room temperature for 1 h and bathed in the primary antibody solutions (1 : 200) at 4°C overnight. The cells were washed three times with PBS the next day, followed by incubation in the dark with Dylight 594 conjugated secondary antibody (1 : 50) at 37°C for 1 h. Then, the cells were washed with PBS for three times and stained with 4,6-diamino-2-phenyl indole (DAPI) for 5 min. The DAPI was removed, followed by another three times of PBS wash. Finally, the cells were bathed with 80% glycerin and used for immunofluorescent image taken under a fluorescent microscope or confocal laser scanning microscope. The dilution rate was 1 : 10000 for DAPI. These results were repeated in at least three independent experiments.

### 2.16. Ectopic Bone Formation

MEFs were transfected with Ad-GFP, Ad-BMP9, Ad-BMP9+Ad-MEG3, and Ad-BMP9+Ad-siMEG3. Twenty-four hours after transfection, cells were collected and resuspended in cold PBS for subcutaneous transplantation (5 × 10^6^ per injection) at the flanks of athymic nude mice (six mice each group, four-six weeks old, and females). Four weeks after transplantation, the mice were euthanized, and the ectopic bone tissues were retrieved for *μ*CT assessment and histological analysis. No bone masses were found in Ad-GFP-transfected group. The nude mice were all ordered from Animal Centre of Chongqing Medical University (Chongqing, China). The animal experiments were approved by the Ethics Committee of Chongqing Medical University.

### 2.17. Histological Staining

The retrieved ectopic bone tissues were fixed in 4% paraformaldehyde for 1 week, decalcified using EDTA solution (pH 7.2) for 4 weeks, dehydrated by ethanol, and embedded with paraffin. Then, the paraffin-covered masses were sliced and subjected to immunofluorescent staining, hematoxylin-eosin (H&E) staining, and Masson's trichrome staining after deparaffinization and rehydration.

### 2.18. Microcomputed Tomographic Analysis and 3D Reconstruction

The harvested samples of bone tissue were scanned with *μ*CT (VivaCT 40, SCANCO MEDICAL AG, Switzerland). The three-dimensional reconstructions and analysis were performed following the software linked to the scanner (*μ*CT 516.1).

### 2.19. NIH ImageJ for Quantifications

The NIH ImageJ (http://rsbweb.nih.gov/ij/download.html) software was used for quantifications. For western blot results, the ratio of target bands to their GAPDH/COXIV was normalized to control, followed by further statistical analysis. For ALP results, the images of each well were measured for further statistical analysis. For histological evaluations, such as IHC, H&E, and Masson's trichrome staining, the positive area percentage was used for quantification and statistical analysis.

### 2.20. Statistical Analysis

The software GraphPad Prism 8 was used to analyze the mean ± SD values and the significant differences between two groups by means of two-tailed Student's *t*-test. *p* values less than 0.05 were considered statistically significant, and *p* values less than 0.01 were considered remarkably significant.

## 3. Results

### 3.1. Effects of MEG3 on BMP9-Induced Osteogenic Differentiation in MEFs

**Figure 1:**
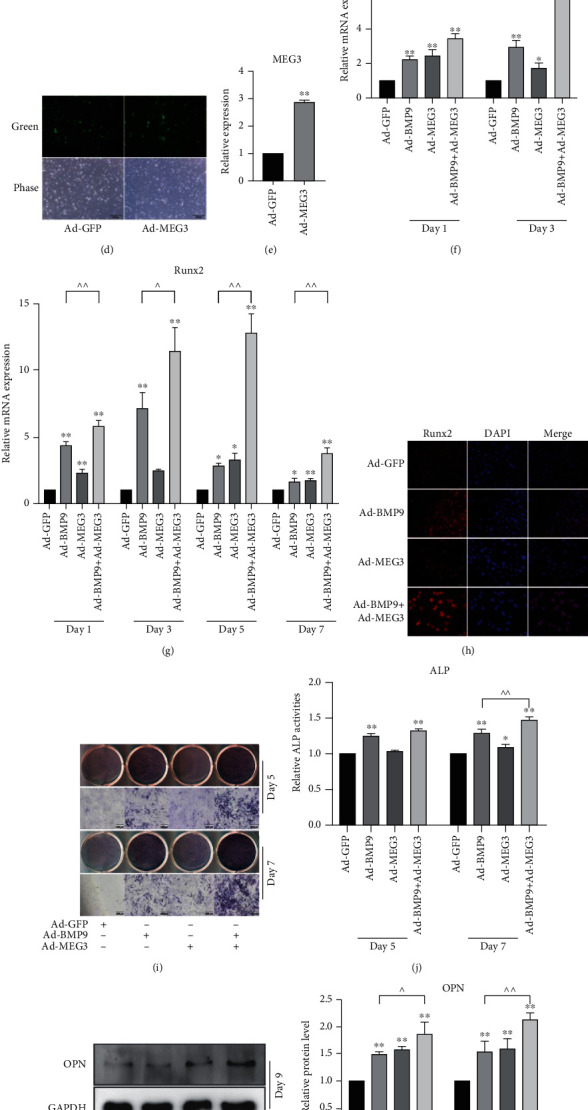
Effects of MEG3 on BMP9-induced osteogenic differentiation in MEFs: (a) RNA-seq analysis showed the different expressions of MEG3 in MEFs and osteogenesis-resistant MEFs; (b) qPCR results showed the endogenous expression of MEG3 in different osteogenic cells; (c) qPCR results demonstrated the effect of BMP9 on the expression of MEG3, ^∗∗^*p* < 0.01 vs. Ad-GFP group; (d) MEFs transfected with Ad-GFP or Ad-MEG3 after 24 h; (e) qPCR results showed the effect of Ad-MEG3 on the expression of MEG3, ^∗∗^*p* < 0.01 vs. Ad-GFP group; (f) qPCR results showed the effect of MEG3 on BMP9-induced mRNA expression of Dlx-5, ^∗^*p* < 0.05, ^∗∗^*p* < 0.01 vs. corresponding Ad-GFP group, ^^*p* < 0.01; (g) qPCR results demonstrated the effect of MEG3 on BMP9-induced mRNA expression of Runx2, ^∗^*p* < 0.05, ^∗∗^*p* < 0.01 vs. corresponding Ad-GFP group; ^*p* < 0.05, ^^*p* < 0.01; (h) Immunofluorescent staining results showed the effect of MEG3 on BMP9-induced protein expression of Runx2; (i) ALP staining results demonstrated the effect of MEG3 on BMP9-induced ALP activities; (j) Quantification of ALP staining results demonstrated the effect of MEG3 on BMP9-induced ALP activities after 5 and 7 days, ^∗^*p* < 0.05, ^∗∗^*p* < 0.01 vs. corresponding Ad-GFP group, ^^*p* < 0.01; (k) Western blot results showed the effect of MEG3 on BMP9-induced protein level of OPN; (l) Quantification of western blot results showed the effect of MEG3 on BMP9-induced protein level of OPN, ^∗∗^*p* < 0.01 vs. corresponding Ad-GFP group; ^*p* < 0.05, ^^*p* < 0.01. (m) Alizarin Red S staining results showed the effect of MEG3 on BMP9-induced matrix mineralization; (n) Quantification of Alizarin Red S staining results showed the effect of MEG3 on BMP9-induced matrix mineralization, ^∗∗^*p* < 0.01 vs. Ad-GFP group, ^*p* < 0.05. OR MEFs: osteogenesis-resistant MEFs; Ad-GFP: adenovirus-mediated green fluorescent protein; OPN: osteopontin.

OR MEFs were more difficult to be induced into osteogenic progenitor cells and osteoblasts. The RNA-seq analysis between MEFs and OR MEFs showed that the expression of MEG3 was lower in OR MEFs (Figure 1(a)), and MEG3 was detectable in most osteogenesis-related cell lines (Figure 1(b)). The expression of MEG3 showed a positive correlation with BMP9 (Figure 1(c)). Some studies report the positive osteogenic effect of MEG3, while other studies show the opposite effect. Therefore, we constructed the recombinant adenovirus for overexpression of MEG3 (Figures 1(d) and 1(e)) and investigated the effect of MEG3 on osteogenic differentiation. By recruiting BMP9-induced osteogenic differentiation in MEFs as an *in vitro* osteogenic model, we found that MEG3 potentiated the level of early osteogenic markers, such as Dlx-5 and RUNX2 (Figures 1(f) –1(h)). The same results were obtained by ALP staining (Figures 1(i) and 1(j)), western blot of late osteogenic marker OPN (Figures 1(k) and 1(l)), and matrix mineralization (Figures 1(m) and 1(n)). These results indicate that the osteogenic capability of BMP9 can be enhanced by MEG3.

### 3.2. Effects of Knockdown of MEG3 on BMP9-Induced Osteogenic Differentiation in MEFs

**Figure 2:**
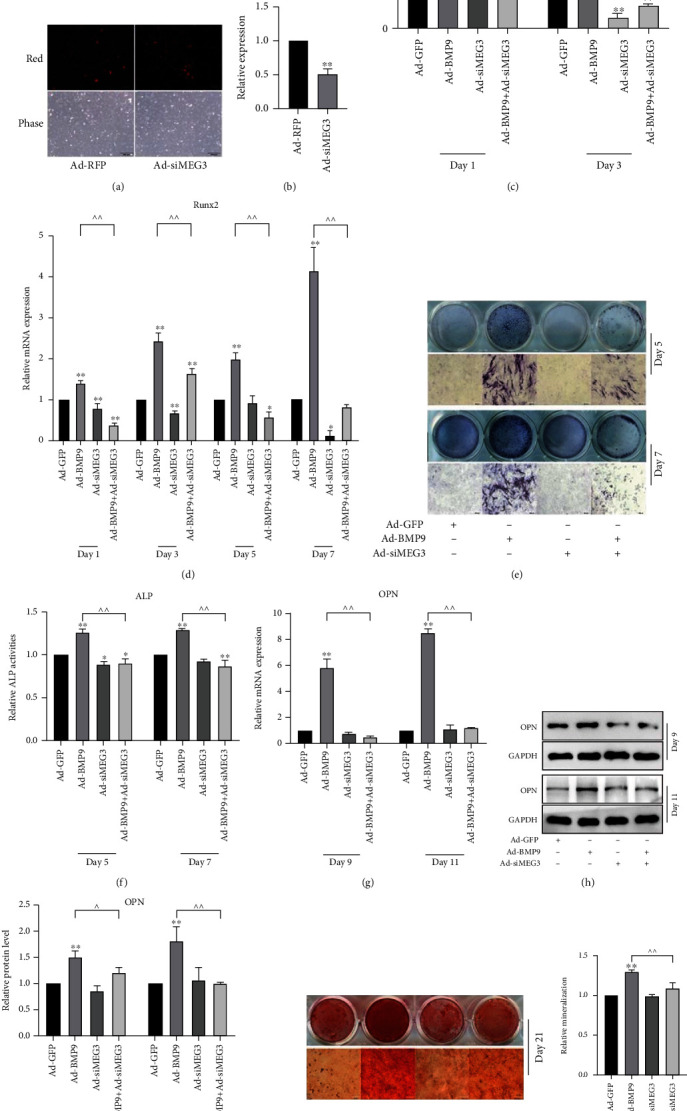
Effects of siMEG3 on BMP9-induced osteogenic differentiation in MEFs: (a) MEFs transfected with Ad-RFP or Ad-siMEG3 after 24 h; (b) qPCR results showed the effect of Ad-siMEG3 on the expression of MEG3, ^∗∗^*p* < 0.01 vs. RFP group; (c) qPCR results showed the effect of siMEG3 on BMP9-induced mRNA expression of Dlx-5, ^∗∗^*p* < 0.01 vs. corresponding Ad-GFP group, ^^*p* < 0.01; (d) qPCR results demonstrated the effect of siMEG3 on BMP9-induced mRNA expression of Runx2, ^∗^*p* < 0.05, ^∗∗^*p* < 0.01 vs. corresponding Ad-GFP group, ^^*p* < 0.01; (e) ALP staining results demonstrated the effect of siMEG3 on BMP9-induced ALP activities after 5 and 7 days; (f) Quantification of ALP staining results demonstrated the effect of siMEG3 on BMP9-induced ALP activities after 5 and 7 days, ^∗^*p* < 0.05, ^∗∗^*p* < 0.01 vs. corresponding Ad-GFP group, ^^p <0.01; (g) qPCR results showed the effect of siMEG3 on BMP9-induced mRNA level of OPN, ^∗∗^*p* < 0.01 vs. corresponding Ad-GFP group, ^^*p* < 0.01; (h) Western blot results showed the effect of siMEG3 on BMP9-induced protein level of OPN; (i) Quantification of western blot results showed the effect of siMEG3 on BMP9-induced protein level of OPN, ^∗∗^*p* < 0.01 vs. corresponding Ad-GFP group, ^*p* < 0.05, ^^*p* < 0.01; (j) Alizarin Red S staining results showed the effect of siMEG3 on BMP9-induced matrix mineralization; (k) Quantification of Alizarin Red S staining results showed the effect of siMEG3 on BMP9-induced matrix mineralization, ^∗∗^*p* < 0.01 vs. Ad-GFP group, ^^*p* < 0.01. Ad-RFP: adenovirus-mediated red fluorescent protein; OPN: osteopontin.

To investigate the opposite effect of MEG3 on BMP9-induced osteogenic differentiation, we constructed the recombinant adenovirus for knockdown of MEG3 (Figures 2(a) and 2(b)). The combined use of BMP9 and siMEG3 showed lower Dlx-5 and RUNX2 mRNA expressions compared with single use of BMP9 (Figures 2(c) and 2(d)). In addition, siMEG3 also decreased BMP9-induced ALP activities (Figures 2(e) and 2(f)), OPN mRNA level (Figure 2(g)) and protein expression (Figures 2(h) and 2(i)), and matrix mineralization (Figures 2(j) and 2(k)). These data suggest that knockdown of MEG3 may lower the osteogenic effect of BMP9 in MEFs.

### 3.3. Effects of MEG3 on BMP9-Induced Ectopic Bone Formation

**Figure 3:**
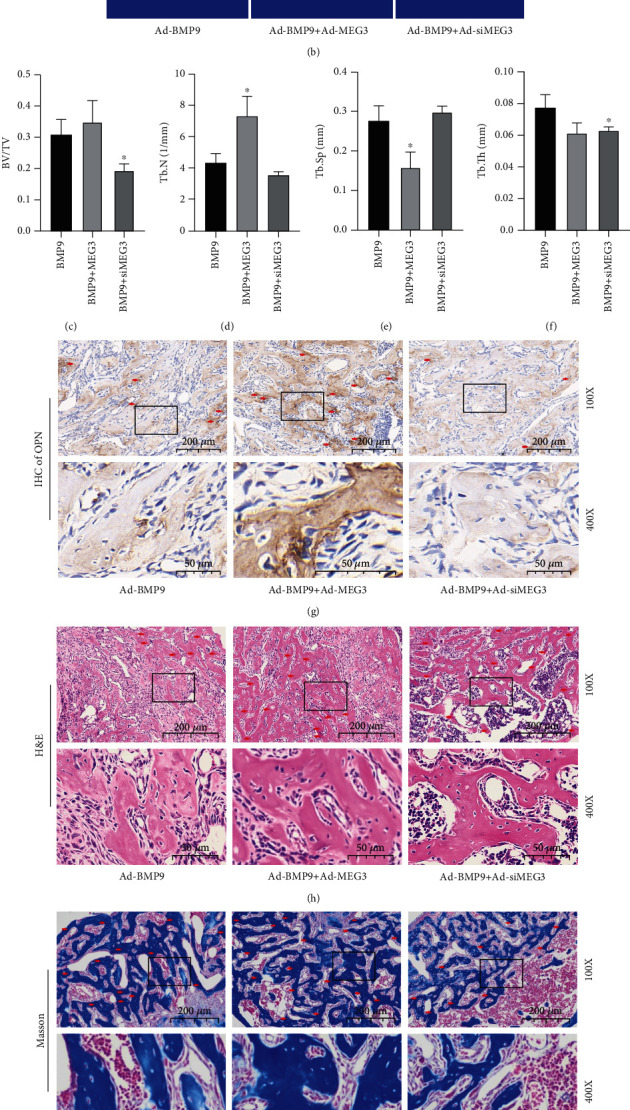
Effects of MEG3 on BMP9-induced ectopic bone formation *in vivo*. (a) Ectopic bone tissues from corresponding nude mice; (b) *μ*CT scan showed the effect of MEG3 on BMP9-induced ectopic bone formation; (c–f) *μ*CT analysis of retrieved ectopic bone tissues, ^∗^*p* < 0.05 vs. corresponding BMP9 group; (g) Immunohistochemical staining of OPN showed the effect of MEG3 on BMP9-induced late osteogenic marker; (h) Hematoxylin-eosin staining showed the effect of MEG3 on BMP9-induced osteoid tissues; (i) Masson's trichrome staining results demonstrated the effect of MEG3 on BMP9-induced osteoid tissues; (j–l) Quantitative analysis of IHC, H&E, and Masson's trichrome staining results demonstrated the effect of MEG3 on BMP9-induced bone formation, ^∗^*p* < 0.05 vs. corresponding BMP9 group, ^∗∗^*p* < 0.01 vs. corresponding BMP9 group. BV/TV: ratio of bone volume to total volume; Tb. N: trabecular number; Tb. Sp: trabecular separation; Tb. Th: trabecular thickness. BMP9, MEG3, and siMEG3 in (c–f) and (j–l) were short for Ad-BMP9, Ad-MEG3, and Ad-siMEG3, respectively.

We next explored the effect of MEG3 on BMP9-induced osteogenic differentiation *in vivo*. By ectopic bone formation, we found an increased mean volume when BMP9 was combined with MEG3, and a decreased mean volume when BMP9 was combined with siMEG3 (Figure 3(a)). The three-dimensional reconstruction of *μ*CT scan (Figure 3(b)) and analysis (Figures 3(c)–3(f)) showed that MEG3 enhanced the ratio of bone volume to total volume and trabecular number while attenuated the trabecular separations. IHC staining showed that MEG3 enhanced BMP9-induced OPN expression *in vivo* while siMEG3 attenuated it (Figure 3(g)). The same effects on bone trabecular and collagen were found by H&E staining and Masson's trichrome staining (Figures 3(h) and 3(i)). These histological evaluations were confirmed by quantifications (Figures 3(j)–3(l)) These data indicate that BMP9-induced osteogenic differentiation *in vivo* can be promoted by MEG3 and attenuated by knockdown of MEG3.

### 3.4. Effects of MEG3 on GSK-3*β*/*β*-Catenin Axis in MEFs

**Figure 4:**
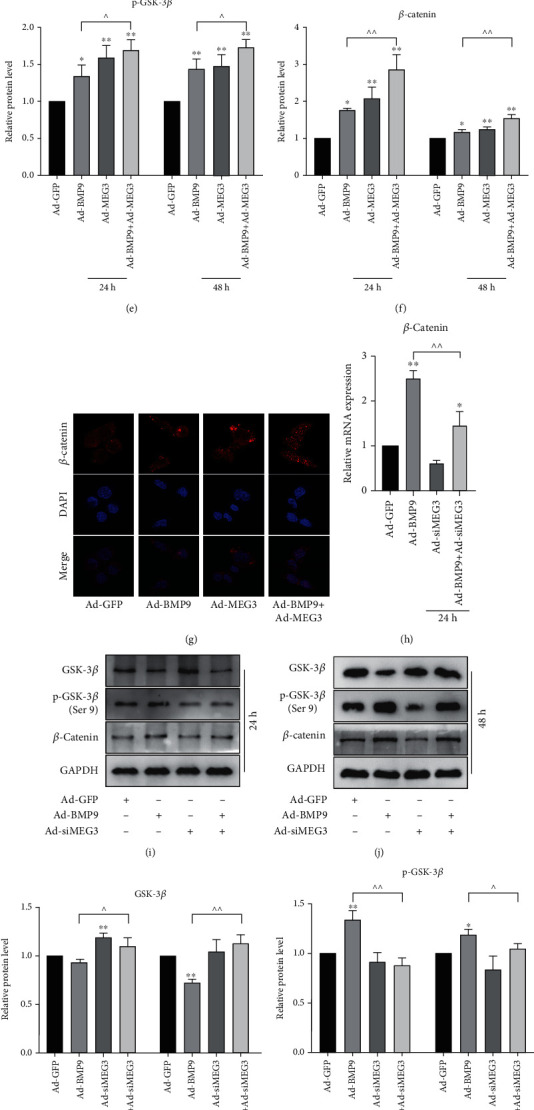
Effects of MEG3 on GSK-3*β*/*β*-catenin axis in MEFs: (a) Immunohistochemical staining showed the effect of MEG3 on the protein expression of *β*-catenin *in vivo*; (b–c) Western blot results showed the effect of MEG3 on the expression of GSK-3*β*, *β*-catenin and the phosphorylation of GSK-3*β* after 24 h and 48 h; (d–f) Quantifications of western blot results from (b) and (c), ^∗^*p* < 0.05, ^∗∗^*p* < 0.01 vs. corresponding Ad-GFP group, ^*p* < 0.05, ^^*p* < 0.01; (g) Confocal laser scanning results showed the effect of MEG3 on the protein expression and the translocation into nucleus of *β*-catenin; (h) qPCR results showed the effect of siMEG3 on the mRNA expression of *β*-catenin, ^∗^*p* < 0.05, ^∗∗^*p* < 0.01 vs. corresponding Ad-GFP group, ^^*p* < 0.01; (i–j) Western blot results showed the effect of siMEG3 on the expression of GSK-3*β*, *β*-catenin and the phosphorylation of GSK-3*β* after 24 h and 48 h; (k–m) Quantifications of western blot results from (i) and (j), ^∗^*p* < 0.05, ^∗∗^*p* < 0.01 vs. corresponding Ad-GFP group, ^*p* < 0.05, ^^*p* < 0.01. IHC: immunohistochemical staining; Ad-GFP: adenovirus-mediated green fluorescent protein.

We next sought to explore the mechanism of the positive osteogenic effect of MEG3. IHC staining showed that MEG3 increased the protein level of *β*-catenin in BMP9-induced ectopic bone tissues (Figure 4(a)). Western blot results showed that MEG3 increased the phosphorylation of GSK-3*β* and the expression of *β*-catenin in BMP9-induced MEFs (Figures 4(b)–4(f)). Confocal laser scanning showed that MEG3 facilitated the translocation of *β*-catenin into nucleus (Figure 4(g)). On the contrary, siMEG3 lowered the phosphorylation of GSK-3*β* and the expression of *β*-catenin (Figures 4(h)–4(m)). These results suggest that MEG3 may potentiate the osteogenic differentiation by enhancing GSK-3*β*/*β*-catenin axis.

### 3.5. Effects of PDK4 on Osteogenic Differentiation and GSK-3*β*/*β*-Catenin Axis in MEFs

**Figure 5:**
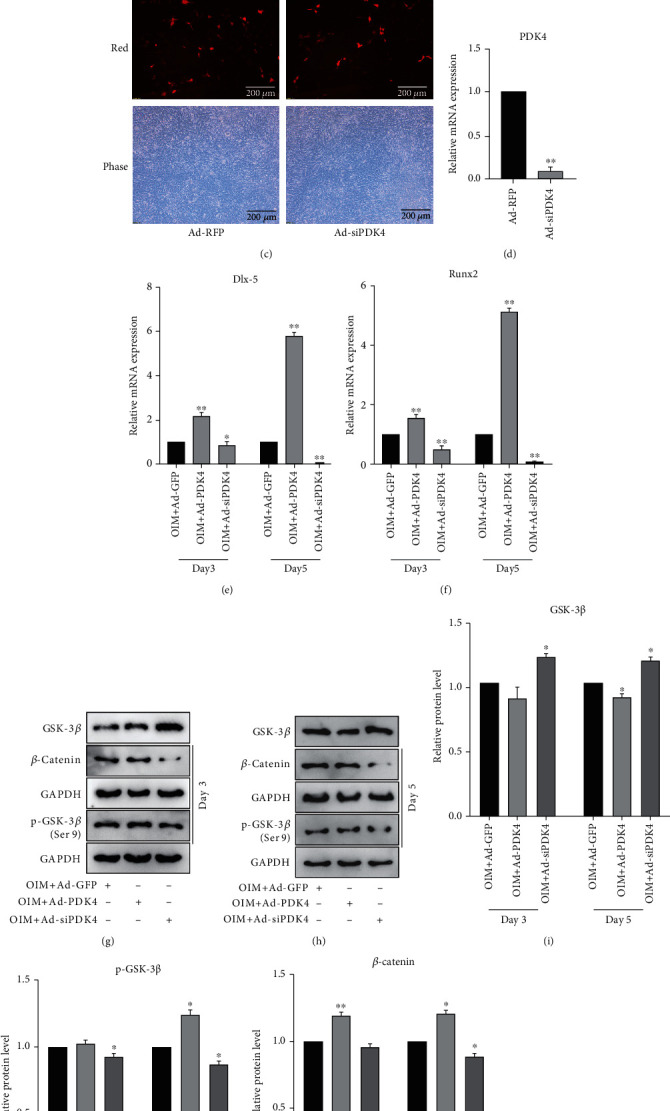
Effects of PDK4 on osteogenesis and GSK-3*β*/*β*-catenin axis in MEFs. (a) MEFs transfected with Ad-GFP or Ad-PDK4 after 24 h; (b) qPCR results demonstrated the effect of Ad-PDK4 on the mRNA level of PDK4, ^∗∗^*p* < 0.01 vs. Ad-GFP group; (c) MEFs transfected with Ad-RFP or Ad-siPDK4 after 24 h; (d) qPCR results demonstrated the effect of Ad-siPDK4 on the mRNA level of PDK4, ^∗∗^*p* < 0.01 vs. Ad-RFP group; (e) qPCR results showed the effect of PDK4 on OIM-induced mRNA level of Dlx-5 after 3 and 5 days, ^∗^*p* < 0.05, ^∗∗^*p* < 0.01 vs. corresponding OIM + Ad-GFP group; (f) qPCR results showed the effect of PDK4 on OIM-induced mRNA level of Runx2, ^∗∗^*p* < 0.01 vs. corresponding OIM + Ad-GFP group; (g–h) Western blot results showed the effect of PDK4 on the expression of GSK-3*β*, *β*-catenin and the phosphorylation of GSK-3*β* after 3 and 5 days; (i–k) Quantifications of western blot results from (g) and (h), ^∗^*p* < 0.05, ^∗∗^*p* < 0.01 vs. corresponding OIM + Ad-GFP group; (l) Coimmunoprecipitation results showed the combination of PDK4 and p-GSK-3*β*. Ad-GFP: adenovirus-mediated green fluorescent protein; Ad-RFP: adenovirus-mediated red fluorescent protein; OIM: osteogenic inductive medium.

As PDK4 is involved in vascular calcification and osteogenic differentiation, we constructed recombinant adenoviruses for overexpression and knockdown of PDK4 (Figures 5(a)–5(d)). OIM culture showed that PDK4 potentiated the mRNA level of early osteogenic marker Dlx-5 and RUNX2 (Figures 5(e) and 5(f)). Western blot results demonstrated that PDK4 also enhanced the phosphorylation of GSK-3*β* and the protein level of *β*-catenin (Figures 5(g) and 5(h)), which was confirmed by quantification and statistical analysis (Figures 5(i)–5(k)). Further co-IP results showed that PDK4 combined with phosphorylated GSK-3*β* (Figure 5(l)). These data suggest that PDK4 may enhance GSK-3*β*/*β*-catenin axis by promoting the phosphorylation and degradation of GSK-3*β*.

### 3.6. Effects of MEG3 on PDK4 and Micro RNAs in MEFs

**Figure 6:**
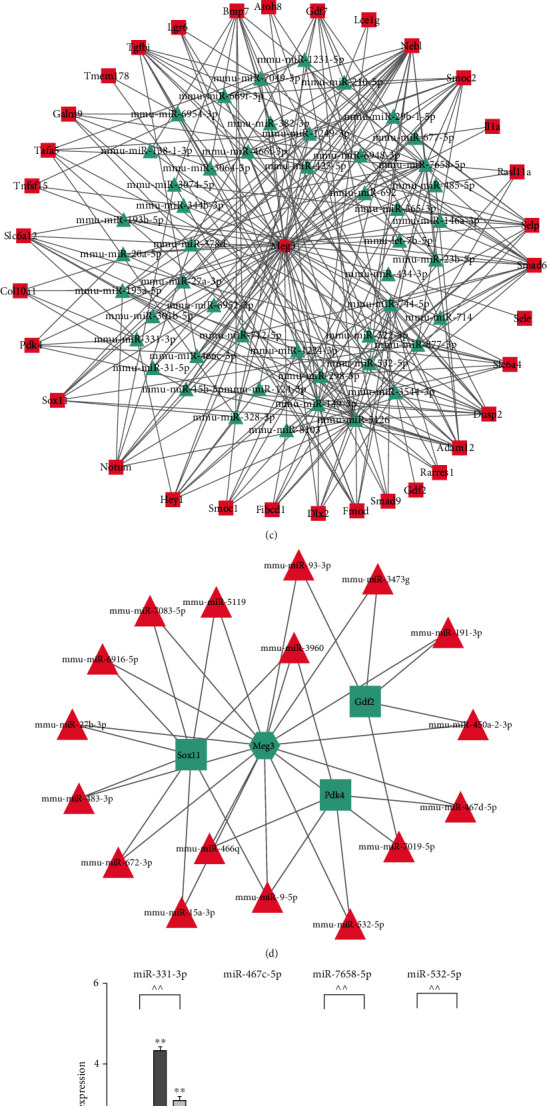
Effects of MEG3 on PDK4 and microRNAs in MEFs. (a) qPCR results showed the effect of overexpression of MEG3 on the mRNA expression of PDK4 after 1 and 2 days, ^∗^*p* < 0.05, ^∗∗^*p* < 0.01 vs. corresponding Ad-GFP group, ^^*p* < 0.01; (b) qPCR results showed the effect of siMEG3 on the mRNA expression of PDK4 after 1 and 2 days, ^∗^*p* < 0.05, ^∗∗^*p* < 0.01 vs. corresponding Ad-GFP group; ^^*p* < 0.01; (c–d) The lncRNA-miRNA-mRNA genes formed a ceRNA network in which the upregulated genes were shown in red, and the downregulated genes were shown in green, the triangle, square, and oval represent miRNAs, mRNAs, and lncRNAs, respectively; (e) qPCR results showed the effect of MEG3 on the expression of microRNAs possibly involved in the effect of MEG3 on PDK4, ^∗∗^*p* < 0.01 vs. corresponding Ad-GFP group; ^^*p* < 0.01. Ad-GFP, adenovirus-mediated green fluorescent protein.

MEG3 and PDK4 showed the same positive effect on GSK-3*β*/*β*-catenin axis. Therefore, we investigated the relationship between MEG3 and PDK4. qPCR results showed that MEG3 increased the mRNA level of PDK4 with or without the interference of BMP9 (Figure 6(a)). However, knockdown of MEG3 decreased the mRNA level of PDK4 (Figure 6(b)). Given that lncRNA can act as a ceRNA to sponge miRNA, we constructed the ceRNA network of MEG3 based on the target gene of miRNA and the expression correlation (Figures 6(c) and 6(d)), in which the mRNAs (including PDK4) showed a positive correlation with MEG3, while the miRNAs showed a negative correlation with MEG3. We selected four miRNAs (miR-331-3p, miR-467c-5p, miR-7658-5p, and miR-532-5p) that involved in the effect of MEG3 on PDK4 for further investigation. qPCR results showed that miR-467c-5p, miR-7658-5p, and miR-532-5p may participate in the sponge activity of MEG3 (Figure 6(e)). We selected the most significant miRNA, miR-532-5p for the following experiments. These data suggest that MEG3 may enhance the expression of PDK4 by sponging certain miRNA.

### 3.7. Effects of miR-532-5p on MEG3-Mediated GSK-3*β*/*β*-Catenin Axis and PDK4 Expression in MEFs

**Figure 7:**
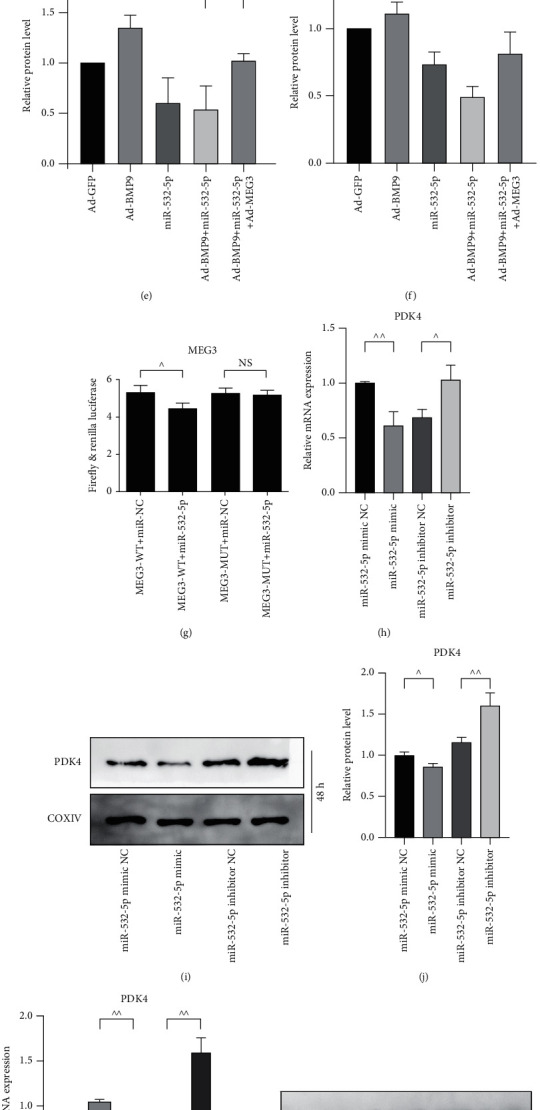
Effects of miR-532-5p on MEG3 and PDK4 in MEFs. (a) qPCR results showed the effect of overexpression of MEG3 on the expression of miR-532-5p after 1 and 2 days, ^∗∗^*p* < 0.01 vs. corresponding Ad-GFP group, ^^*p* < 0.01; (b) qPCR results showed the effect of siMEG3 on the expression of miR-532-5p, ^∗^*p* < 0.05, ^∗∗^*p* < 0.01 vs. corresponding Ad-GFP group, ^^*p* < 0.01; (c) Western blot results demonstrated the effect of miR-532-5p on MEG3-medicated GSK-3*β*/*β*-catenin axis; (d–f) Quantifications of western blot results from (c), ^*p* < 0.05, ^^*p* < 0.01; (g) Dual luciferase reporter results showed the effect of miR-532-5p and MEG3-WT/MEG3-MUT on the transcriptional activity of MEG3, ^*p* < 0.05; (h) qPCR results showed the effect of miR-532-5p on the mRNA expression of PDK4, ^*p* < 0.05, ^^*p* < 0.01; (i) Western blot results demonstrated the effect of miR-532-5p on the protein level of PDK4; (j) Quantification of western blot results from (i), ^*p* < 0.05, ^^*p* < 0.01; (k) qPCR results showed the effect of MEG3 and miR-532-5p on the mRNA level of PDK4, ^^*p* < 0.01; (l) Western blot results showed the effect of MEG3 and miR-532-5p on the protein expression of PDK4; (m) Quantification of western blot results from (l), ^*p* < 0.05. Ad-GFP: adenovirus-mediated green fluorescent protein; NS: no significance; COXIV: cytochrome C oxidase IV.

**Figure 8:**
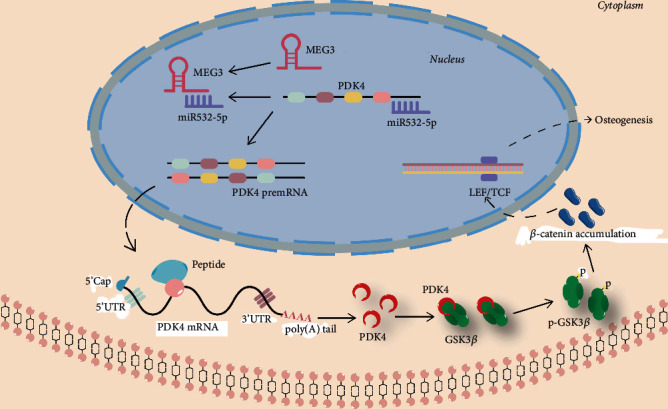
Possible mechanism of the effects of MEG3 on PDK4 and osteogenesis.

Based on the previous results, we further detected the effect of MEG3 on the expression of miR-532-5p. The qPCR results showed that MEG3 inhibited the expression of miR-532-5p while knockdown of MEG3 promoted this expression with or without the participation of BMP9 (Figures 7(a) and 7(b)). Western blot results demonstrated that miR-532-5p inhibited the phosphorylation of GSK-3*β* and the protein level of *β*-catenin (Figures 7(c)–7(f)). However, this effect was partly reversed by the combination of MEG3. For further validation, we constructed the MEG3-wild type (WT) and MEG3-mutation (MUT) in psicheck2 vector (Figure S1). The dual luciferase reporter results demonstrated that miR-532-5p attenuated the transcriptional activity of MEG3 with the combination of MEG3-WT but not the combination of MEG3-MUT (Figure 7(g)). Finally, we sought to explore the relationship between miR-532-5p and PDK4 as well as the role of miR-532-5p in MEG3-regulated PDK4 expression. The qPCR and western blot results revealed that miR-532-5p mimic attenuated the mRNA and protein level of PDK4, while miR-532-5p inhibitor showed the opposite effect (Figures 7(h)–7(j)). Accordingly, miR-532-5p mimic lowered MEG3-promoted mRNA and protein level of PDK4; miR-532-5p inhibitor promoted the positive effect of MEG3 on the expression of PDK4 (Figures 7(k)–7(m)). These data imply that MEG3 attenuated the expression of miR-532-5p. In addition, MEG3-promoted PDK4 and GSK-3*β*/*β*-catenin axis can be negatively regulated by miR-532-5p. The possible mechanism of MEG3 on PDK4 and osteogenesis was demonstrated in Figure 8.

## 4. Discussion

In this study, we investigated the effect of lncRNA MEG3 on osteogenic differentiation in MSCs and demonstrated the possible mechanism based on the experimental results. We found that MEG3 enhanced the osteogenic potential of BMP9 both *in vitro* and *in vivo*, while knockdown of MEG3 attenuated BMP9-induced osteogenic differentiation. Overexpression of MEG3 and PDK4 both increased the phosphorylation of GSK-3*β* and the protein level of *β*-catenin. Moreover, MEG3 promoted the mRNA level of PDK4, indicating that MEG3 may potentiate osteogenesis via PDK4-mediated GSK-3*β*/*β*-catenin pathway. The ceRNA network showed that miR-532-5p was involved in the effect of MEG3 on PDK4, which was confirmed by dual luciferase reporter and the recruitment of miRNA mimic and inhibitor. Hence, our study suggests that MEG3 may promote PDK4-mediated osteogenic differentiation of GSK-3*β*/*β*-catenin by acting as a ceRNA, sponging miR-532-5p, and offsetting the inhibition of miR-532-5p to PDK4.

As a kind of lncRNA, MEG3 has been widely regarded as a tumor suppressor and studied in cancer research, such as squamous cell carcinoma, colorectal cancer, and glioblastoma [5, 6, 33]. It has been reported that MEG3 is essential for stimulation of the tumor suppressor p53 pathway [34]. In recent years, increasing evidence has revealed the osteogenic correlation of MEG3, although the studies contradict with each other. Our results showed the positive osteogenic effect of MEG3 on osteogenic model in MEFs (Figures 1(i)–1(n) and Figures 3(g)–3(i)), which was in accordance with previous studies [7, 8, 35]. However, there are studies reporting the inhibition of MEG3 on osteogenic differentiation [36]. The study demonstrates that the expressions of MEG3 in MSCs from postmenopausal women with osteoporosis (PMOP) and ovariectomized (OVX) mice are significantly higher than that of their healthy controls, respectively. This is probably because of the pathological state of MSCs. As we know, unlike glucocorticoid-induced osteoporosis (GIOP) with dramatically reduced osteogenesis, PMOP is manifested as high bone turnover [37, 38]. The osteogenic markers, such as OCN, bone ALP, and N-terminal propeptide of type I procollagen (PINP), are often detected higher in PMOP patients than that of healthy women. Thus, we assume that the MSCs taken from PMOP or OVX mice were in a high bone turnover state with high levels of osteogenic markers compared with healthy controls. Although the expressions of MEG3 in these MSCs are also higher than controls, they positively correlated to the expressions of osteogenic markers. This coincidentally explained the cause and effect given that according to our results, MEG3 can enhance the osteogenic potential and the expression of osteogenic markers in MSCs. However, more osteogenesis-related studies on MEG3 are needed to profoundly comprehend the function of MEG3.

It has been reported that PDK4 plays a critical role in vascular calcification [19, 39]. PDK4 increases the phosphorylation of Smad1/5/8 and osteogenic differentiation in vascular smooth muscle cells (VSMCs) [21]. Meanwhile, osteogenic induction of VSMCs also increases the expression of PDK4 [20], indicating that PDK4 is implicated in osteogenic differentiation, which shares similar process with vascular calcification. However, studies on PDK4 and osteogenic differentiation of MSCs are rare. We demonstrated that PDK4 promoted the expressions of osteogenic markers in OIM-induced MSCs (Figures 5(e) and 5(f)), which was in accordance with previous calcification-related studies. Since Wnt/*β*-catenin signaling pathway plays an established role in osteogenesis and impacts on arterial calcification [40–42], we detected the effect of PDK4 on Wnt signaling and found that PDK4 potentiated the phosphorylation of GSK-3*β* and the protein level of *β*-catenin (Figures 5(g)–5(k)). The co-IP results made the relationship between PDK4 and Wnt/*β*-catenin more specific, that PDK4 may combine with GSK-3*β* and potentiate the latter phosphorylation, leading to the accumulation of *β*-catenin (Figure 5(l)). Similarly, MEG3 was also found to enhance the phosphorylation of GSK-3*β*, the protein level (Figures 4(b)–4(f)), and the translocation into the nucleus of *β*-catenin (Figure 4(g)). Previous studies report the opposite effect that MEG3 inhibit Wnt signaling and the expression of *β*-catenin in liver cancer cells and malignant melanoma cells [43, 44]. The inconsistent effects of MEG3 on Wnt signaling in osteogenesis and tumorigenesis may be attributed to different cell lines and different fields. No study has reported the relationship between MEG3 and PDK4 even though they exerted the same positive osteogenic effect in MSCs. Our results showed that MEG3 promoted the expression of PDK4 while knockdown of MEG3 attenuated it (Figures 6(a) and 6(b)). These findings together with the effects of MEG3 and PDK4 on Wnt signaling may form a new sight for lncRNA-mediated osteogenic differentiation.

lncRNAs often act as ceRNAs to regulate osteogenic differentiation and adipogenic differentiation in MSCs via sponging certain miRNAs [12, 45, 46]. Therefore, we constructed a ceRNA network of MEG3 to reveal the possible miRNAs implicated in the effect of MEG3 on PDK4 expression (Figures 6(c) and 6(d)). Although miR-331-3p, miR-467c-5p, miR-7658-5p, and miR-532-5p were all correlated to MEG3 and PDK4, miR-532-5p dramatically decreased once MEG3 was applied (Figure 6(e)). miR-532-5p demonstrates significant correlation with lumbar spine bone mineral density [13] and participates in parathyroid hormone- (PTH-) stimulated expression of matrix metalloproteinase-13 in osteoblasts [47]. These reports together with our results indicate that miR-532-5p may play a key role in bone metabolism. Except for miR-532-5p, MEG3 also decreased the expression of miR-467c-5p and miR-7658-5p. miR-331-3p is a possible sponging target of MEG3 according to the ceRNA network, but our results showed that miR-331-3p was promoted by MEG3. The effects of these miRNAs on osteogenic differentiation and bone metabolism need further exploration.

It is well known that canonical Wnt signaling is based on Wnt-induced phosphorylation, degradation of downstream GSK-3*β*, and the subsequent accumulation and translocation into nucleus of *β*-catenin. Although we investigated the expression of *β*-catenin from whole cell perspective (Figures 5(g) and 5(h)), we failed to check the protein level of *β*-catenin in the cytoplasm and in the nucleus separately, which gains persuasiveness to the effect of PDK4 on *β*-catenin. Similarly, our co-IP experiments failed to immunoprecipitated GSK-3*β* and western blotting PDK4 reversely (Figure 5(l)). Furthermore, the interactions between miR-532-5p and PDK4 were detected by qPCR and western blot (Figures 7(h)–7(j)), but a luciferase reporter assay could be more suitable to confirm the direct or indirect interactions. In further studies, we will perform these experiments to make our results more convincing.

## 5. Conclusion

Our study suggests that MEG3 can positively potentiate the osteogenic differentiation in BMP9-induced osteogenic model. MEG3 may chiefly enhance GSK-3*β*/*β*-catenin axis and osteogenesis via sponging miR-532-5p and the subsequent activation of PDK4.

## Figures and Tables

**Table 1:** Sequences used in qPCR assay.

Genes	Sequences (5′ to 3′)
RUNX2-F	GCCAATCCCTAAGTGTGGCT
RUNX2-R	AACAGAGAGCGAGGGGGTAT
Dxl-5-F	GGCCGCTTTACAGAGAAGGT
Dxl-5-R	GGTGACTGTGGCGAGTTACA
OPN-F	TGCACCCAGATCCTATAGCC
OPN-R	CTCCATCGTCATCATCATCG
*β*-Catenin-F	GTGCAATTCCTGAGCTGACA
*β*-Catenin-R	CTTAAAGATGGCCAGCAAGC
PDK4-F	CTGCCTGACCGCTTAGTGAA
PDK4-R	TGCCTTGAGCCATTGTAGGG
MEG3-F	GGGTGGGGTGCTTCCTTC
MEG3-R	CCTCAGGCTGCTGCAGTT
miR-331-3p-F	AGCCGCCCCTGGGCCTATCCT
miR-331-3p-R	GTGCAGGGTCCGAGGTCCGAG
miR-467c-5p-F	GCGGCGGTAAGTGCGTGCATG
miR-467c-5p-R	GTGCAGGGTCCGAGGTCCGAG
miR-7658-5p-F	GCGGCGGTGTGGGCGTGGCGGTG
miR-7658-5p-R	GTGCAGGGTCCGAGGTCCGAG
miR-532-5p-F	GCGGCGGCATGCCTTGAGTGTAG
miR-532-5p-R	GTGCAGGGTCCGAGGTCCGAG

## Data Availability

The data used to support this study is available from the corresponding authors upon reasonable request.

## Supplementary Materials

Supplementary Materials Figure S1: the vector, MEG3-WT, and MEG3-MUT sequences for dual luciferase reporter.
