# Supplementary material for: lncRNA MEG3 Promotes PDK4/GSK-3β/β-Catenin Axis in MEFs by Targeting miR-532-5p
Source: Oxid Med Cell Longev. 2023 Feb 1;2023:3563663. doi: 10.1155/2023/3563663 (PMC9908332; doi:10.1155/2023/3563663)
Supplement: Supplementary Materials — Figure S1: the vector, MEG3-WT, and MEG3-MUT sequences for dual luciferase reporter. [file 3563663.f1.pdf]

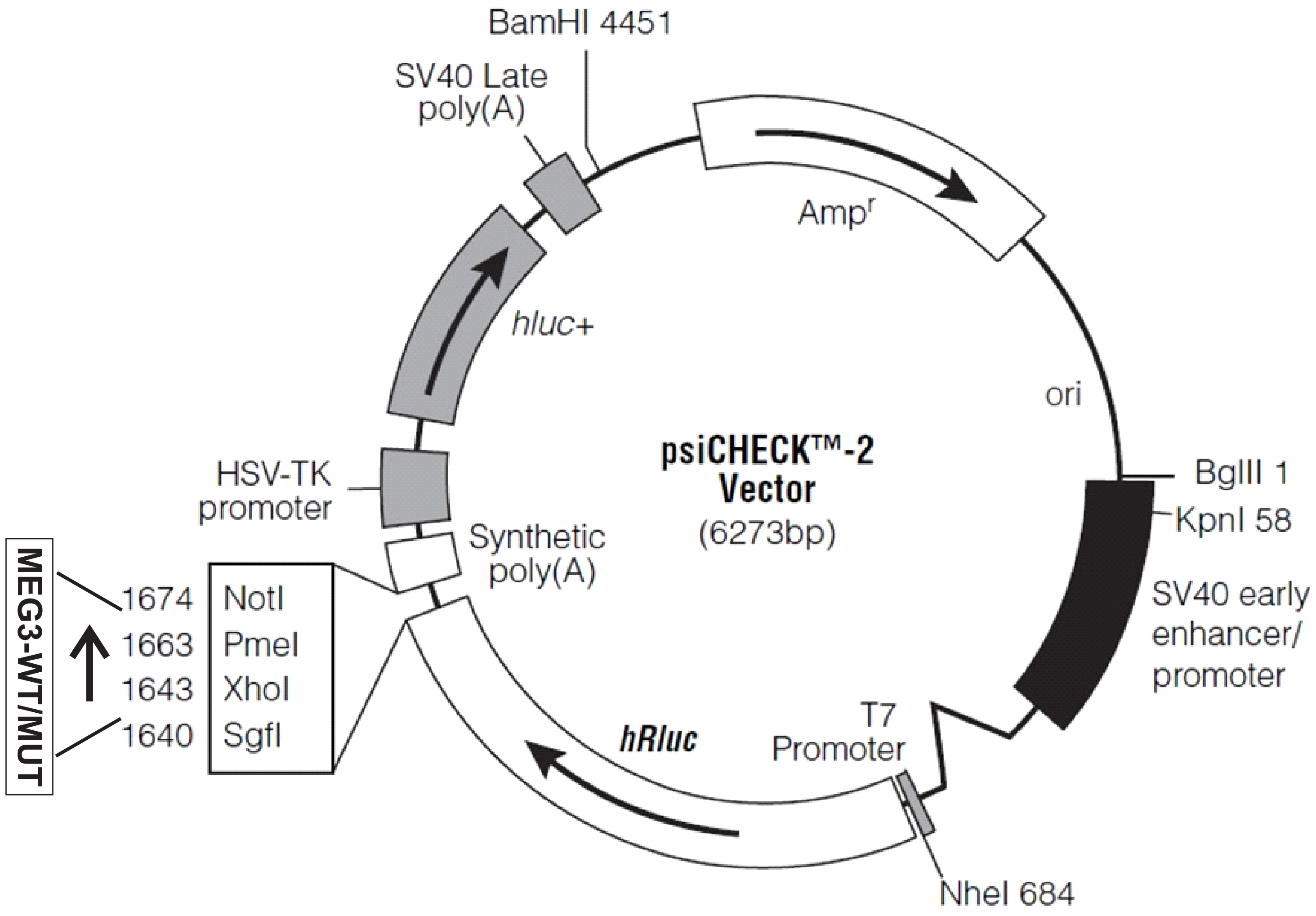

Insertion cite: XhoI and NotI

MEG3-WT in psiCHECK2

gaggacccatgctaggggaggcctcctttcagctttctgctgtgaaggggaagagacgcagggctcttctg
agttctcctcccactagacgcagtaggggcggcaaggccagaacctggagctggacaaattgtgtgatgg
atcttgcctcagggctgttgtgaggattggtataactggacacttctgactgtgaccctaaaggcagtgtatgt
gtccctgggggtgtgccagagcattggaaaccctagccctggagtgggggtggccttg

MEG3-MUT in psiCHECK2

gaggacccatgctaggggaggcctcctttcagctttctgctgtgaaggggaagagacgcagggctcttctg
agttctcctcccactagacgcagtaggggcggcaaggccagaacctggagctggacaaattgtgtgTtCC
aAGtAgGGAGTgCCGtgttgtgaggattggtataactggacacttctgactgtgaccctaaaggcagt
gtatgtgtccctgggggtgtgccagagcattggaaaccctagccctggagtgggggtggccttg
